# Enhancing Resources at the Workplace with Health-Promoting Leadership

**DOI:** 10.3390/ijerph14101264

**Published:** 2017-10-20

**Authors:** Paul Jiménez, Anita Bregenzer, K. Wolfgang Kallus, Bianca Fruhwirth, Verena Wagner-Hartl

**Affiliations:** 1 Department of Psychology, University of Graz, A-8010 Graz, Austria; anita.bregenzer@uni-graz.at (A.B.); wolfgang.kallus@uni-graz.at (K.W.K.); bianca_fruhwirth@hotmail.com (B.F.); 2 Faculty Industrial Technologies, Furtwangen University, Campus Tuttlingen, 78532 Tuttlingen, Germany; verena.wagner-hartl@hs-furtwangen.de

**Keywords:** burnout, health, leadership, resources, stress, working conditions, workplace health promotion

## Abstract

Leaders engaging in health-promoting leadership can influence their employees’ health directly by showing health awareness or indirectly by changing working conditions. With health-promoting leadership, leaders are able to support a healthy working environment by providing resource-oriented working conditions for their employees to support their health. Changing working conditions in a health-supportive way can prevent possible negative consequences from critical working conditions (e.g., burnout risk). The present study examined the relationship between health-promoting leadership and the employees’ resources, stress and burnout. To analyze our proposed model, structural equation modelling was conducted in two samples. The resulting model from the first sample of 228 Austrian workers was cross-validated and could be verified with the second sample (N = 263 Austrian workers). The results supported a model in which health-promoting leadership has a strong direct effect on the employees’ resources and an indirect effect on stress and burnout, which was mediated by resources. The results indicate that health-promoting leadership describes the leaders’ capability and dedication creating the right working conditions for their employees by increasing the employees’ resources at the workplace. This in turn minimizes the risk of experiencing burnout.

## 1. Introduction

Identifying risk factors for burnout is one key aspect for the prevention of burnout (see [1]). These risk factors can be seen either in the person him/herself or in the working conditions of employees. By changing working conditions, burnout can be prevented in a more sustainable way than by changing the employees’ individual behavior [2]. This view is also emphasized in the legal obligations in the European countries where psychosocial risk management is seen as a duty for all employers [3,4]. Working conditions can be improved with the help of the organization and especially with the help of leaders. Leaders are able to positively influence critical working conditions to support a health-promoting workplace [5,6,7] and thus establishing preventive factors for burnout [8,9,10]. As a conclusion of their study, Rigotti et al. [11] point out the importance of this topic and that the prevention of risk factors at the workplace with leadership behavior is still underemphasized in leadership research.

Most studies in the field of leadership and employee burnout focus on preventing burnout with specific leadership skills, such as showing transformational leadership [12,13]. In comparison to this person-focused approach, where the interaction between leader and employee is highlighted, the promotion of healthy working conditions with the support of leadership is increasingly becoming a central focus of more recent research. Studies investigated working conditions as mechanisms that mediate the relationship between leadership behavior and employee burnout [11]. 

The present paper focuses on a concept of health-promoting leadership that includes this approach of creating working conditions to indirectly support employee health [14]. We consider working conditions as a direct outcome of health-promoting leadership behavior [15] as well as a mediator between health-promoting leadership and the employees’ health-related outcomes [11]. Leaders who engage in health-promoting leadership should be able to support a healthy working environment by managing working conditions and changing health-related risk factors. The focus regarding risk factors in the working environment especially concerns burnout, one of the worst results of negative strain outcomes.

Recent research has identified six areas of worklife that are possible risk factors for burnout: workload, control, reward, community, fairness, and values [1,16]. By reducing mismatches between individual and organization in these six areas, a health-promoting workplace can be achieved. The six areas of worklife are a framework that encompasses the most important job stressors that might lead to health-impairments, such as burnout [16]. The areas of worklife can be used to support designing interventions that would improve the working conditions [1]. This view of Maslach and Leiter [1] implies a broader view of working conditions and includes more potential risk factors that could be addressed with the support of leadership.

In the concept of health-promoting leadership, leaders can give their support in creating a health-promoting workplace by changing these six areas [14]. Reducing possible negative consequences from critical working conditions (e.g., burnout risk) can be seen as a preventive approach to secure health at the workplace. This approach can be found in recent models of psychosocial risk assessment [17], where risk factors and their negative outcomes have to be assessed and changed if needed. Negative outcomes of work-related risk factors are often operationalized with stress or burnout (e.g., [18,19]). However, there is more to a healthy workplace than only having low stress or low burnout—a healthy workplace should also focus on promoting resources to improve health (e.g., [20]).

Resources play an important role in reducing stress and burnout. Burnout can be described as a process that results from stress through high work demands and inadequate resources at work [21,22]. Resources at work (e.g., work-related or social resources) are an important factor when it comes to regulate the individual experience of stress and therefore reduce components of burnout, like emotional exhaustion or cynicism [23]. Engaging in health-promoting leadership is found to be positively related to the employee’s perception of resources at the workplace, such as a positive working climate [7]. Leaders can maintain or increase resources by supporting recovery at the workplace. A leader that motivates employees to adopt a healthy lifestyle can positively influence the recovery state of employees, as employees are supported to be more attentive to their individual recovery process and take time to relax and “recharge their batteries”. Next to influencing recovery processes to restore lost resources, leaders might be able to influence workplace resources directly, by creating possibilities to get social support from colleagues or raising decision latitude at the workplace.

The approach of creating working conditions to support the balance between stress and resources and therefore to indirectly support employee health starts to get attention in research about health-promoting leadership. Vincent [24] stated that health-promoting leadership should aim at reducing stressors at the workplace as well as enhancing workplace resources. Rigotti et al. [11] concluded that health-promoting leadership impacts employee health by influencing their resources at work, and to a lesser extent, by influencing stressors. 

Our study aims at extending past research about the effects of health-promoting leadership by investigating the mechanisms between health-promoting leadership, resources, stress, and burnout. We analyze resources and stress not only as outcomes of health-promoting leadership, but also as possible mediators in the relationship between health-promoting leadership and burnout. More specifically, we strongly focus on the balance between resources and stress [23] as a mediator between health-promoting leadership and health-related outcomes (e.g., burnout). Resources and stress have to be investigated together in one model to get more insight in the underlying mechanisms between health-promoting leadership and the employee’s burnout-risk. Especially when investigating the critical effects of stress on burnout, the availability and usage of resources needs to be included to understand if stress has impairing (i.e., burnout) or non-impairing outcomes (i.e., activation). 

The present study contributes to existing research in two different ways: (a) we investigate if leaders can support the balance between resources and stress with their health-promoting behavior and (b) we investigate if leaders can further reduce their employees’ burnout by influencing the employees’ balance between resources and stress.

### 1.1. Health-Promoting Leadership, Employee Health and Burnout

Leader behavior is seen as an important determinant of a healthy workplace. Leader behavior to support employee health can comprise promoting employees’ health directly (person-focused action), promoting healthy work designs (system-focused action), mitigating the impact of environmental stressors (moderating action), cultivating health-related shared perceptions (climate control), and being a role model (modelling) to exemplify particular health behavior [15]. With these five possible pathways, leaders are able to influence employee health, whereas the pathways system-focused action, moderating action and climate control are going beyond the simple leader-employee relationship by including the whole working environment.

Most studies focus on the first pathway where leaders directly promote employee health [12,13,25]. The second pathway where leaders are regarded as promoters of healthy work systems is usually not addressed in leadership research. Nevertheless, there are some studies that especially focus on leadership that supports a working environment by giving control possibilities at work, such as giving opportunities to participate in decisions, involving employees in information sharing and problem solving, or consulting employees before making important decisions [9,26]. 

The concept of health-promoting leadership presented in this article focuses on this second pathway of creating working conditions that support and enhance employee health [11]. The third pathway, were leaders buffer the negative impact of stressors, or amplify organizational and personal resources is included in the concept, as well. Leaders are able to buffer negative effects of working conditions, if the work environment allows them to engage in this buffering behavior. Health-promoting leadership focuses on identifying specific components in leadership behavior that are able to positively influence the working conditions of employees [27]. Health-promoting leadership can be distinguished from other leadership behaviors, such as transformational leadership [27]. Health-promoting leadership is found to be better predictor for employee health than transformational leadership, explaining additional variance in employee health beyond transformational leadership [23,28]. 

Health-promoting leadership considers leader behavior in different working areas, which can be related to the concept of the areas of worklife [1]. According to this concept, burnout occurs because of a chronic mismatch between the person and the working environment. Maslach and Leiter [1] were able to identify six organizational risk factors (areas of worklife), where this mismatch between person and organization can be found: workload, control, reward, community, fairness and values. To prevent burnout, changing these areas of worklife by reducing the mismatch between person and environment is an important task for the organization. In particular, leaders are seen as the most salient persons in the working environment and can be regarded as representatives for the organization’s culture [29]. Therefore, leaders play a major role when it comes to influencing work-related aspects like the areas of worklife. Leaders are able to recognize mismatches between their employees and the working conditions and are able to reduce these mismatches. By considering the six areas of worklife, leaders can arrange the conditions at the workplace in a health-promoting way (see also [1,29]). 

#### 1.1.1. Workload

Leaders are able to offer opportunities to balance the workload by giving enough time to finish the work or giving enough resources to meet the work demands.

#### 1.1.2. Control

Giving an appropriate amount of autonomy, participation, and giving opportunities to use skills and giving possibilities to try out new ideas are only a few examples of how leaders can support their employees.

#### 1.1.3. Reward

Especially reward is an important part of the positive relationship between leader and employee. Achieved work should be appreciated, effort should not go unnoticed, and every input should be valued.

#### 1.1.4. Community

Aspects such as praise, comfort, and humor as well as a respectful cooperation are essential points in a civil community that can be implemented with the help of leadership.

#### 1.1.5. Fairness

The sense of fairness is an individual feeling and therefore it is difficult to generalize. Nevertheless, leadership behavior can follow some basic rules: employees should be treated in a fair manner and all resources should be shared fairly.

#### 1.1.6. Value-Fit

Leaders are able to support consistency of different values by bringing the employees’ and organization’s values in line and arranging tasks that are consistent with personal objectives and those of the company. It is also important to take care that nobody has to work against his/her own values.

In addition to changing working conditions, leaders can reduce burnout-risk by focusing directly on their employees’ health behavior. This more direct approach of health-promoting leadership to lower burnout-risk is the aspect of health awareness. According to Wegge et al. [15], the aspect of health awareness is related to the person-oriented action approach and is highlighted in leadership concepts of Franke et al. [28] and Gurt et al. [7]. Health awareness means that leaders take responsibility for their employees’ health by promoting workplace health promotion projects, communicating about health-relevant topics or simply caring about their employees’ health [7,28].

Health-promoting leadership consists of showing health awareness and changing working conditions (e.g., the areas of worklife: workload, control, reward, community, fairness, value-fit). With health-promoting leadership, leaders are able to establish health-promoting working conditions that are able to reduce the employees’ burnout-risk and enhance employee health. Studies investigating the link between leadership behavior and employee health often found that positive leadership behavior changes the employees’ resources at the workplace (e.g., job control, role clarity, possibilities for development, person-environment fit; see also [25,30,31]. The same applies to health-promoting leadership that aims to change the employees’ working conditions in a way that they are resources to the employees [27]. 

Health-promoting leadership and its underlying seven dimensions can be assessed with the questionnaire health-promoting leadership conditions (HPLC). In the HPLC, employees rate the frequency of their leaders’ health-promoting behavior. Their answers allow conclusions about the health-promoting design of the working conditions. 

### 1.2. Burnout as a Result of an Imbalance of Stress and Resources

In addition to leadership and organizational risk factors, another important factor in identifying the burnout risk is the imbalance between stress and resources. A concept that specifically highlights the important role of resources is the job demands-resources model [21]. According to this model, negative outcomes of stress (such as burnout) can be decreased if resources are high. Vice-versa, low resources might lead to exhaustion and low engagement and eventually results in burnout.

The job demands-resources model strongly focus on job demands as external influences, described as physical, mental, social or organizational aspects of the job [32]. External influences are difficult to measure, as they can be either negative or positive, depending on the subjective appraisal of the person [33]. On the other hand, the subjective appraisal of the demands as well as the subjective reaction to the demands is less ambiguous and can be assessed more precisely, particularly when assessing at the individual level [34]. The most researched subjective appraisal in research about organizational risk factors would be stress, that can be defined as the inner state of the person, which is caused as a reaction to external stressors [35].

A concept that refers to the imbalance of stress and resources is the model of the recovery-stress-balance [23]. In this model, resources are seen as the recovered resources that have been restored in recovery processes. This view is also stated by Oerlemans et al. [36] and Zijlstra et al. [37], where resources have to be restored regularly to show stress-reducing effects. According to the model of the recovery-stress-balance [23], prolonged stressful situations might lead to negative long-term effects such as burnout if resources have not been replenished in recovery processes. Regulating the balance between stress and resources by developing successful recovery strategies can cancel this negative process [23,38]. The concept of the recovery-stress-balance focuses on the mechanisms of stress and resources which lead to possible burnout risk and is similar to the effort-recovery model [22], where the critical effect of stress on burnout can be prevented by recovery, which stabilizes the psycho-biological system to a specific baseline level. 

Especially resources on the task level (decision latitude and autonomy) and on the interpersonal level (social support from colleagues) have been found to reduce stress and burnout [39,40,41]. In the present study, we conceptualize resources as a combination of task level and interpersonal level resources, which have been restored in recovery processes.

### 1.3. Theoretical Model and Hypotheses

The aim of the present study is to investigate the paths between health-promoting leadership, resources, stress and burnout. Health-promoting leaders are able to increase the resources of their employees by changing working conditions in a health-promoting way [27]. By changing working conditions, leaders support their employees by providing them with resources to adapt to the demands of work. Therefore, we propose that a high level of health-promoting leadership is positively related to resources and negatively related to stress. This leads to following hypotheses:

**Hypothesis** **1.** Health-promoting leadership has a positive relationship with resources.

**Hypothesis** **2.** Health-promoting leadership has a negative relationship with stress.

In line with the model of the recovery-stress balance [23], burnout is seen as a process that results from an imbalance between stress and resources. More specifically, the model of the recovery-stress balance proposes that resources reduce the negative effect of stress on burnout.

**Hypothesis** **3.** Stress has a positive relationship with the experience of burnout.

**Hypothesis** **4.** Resources have a negative relationship with the experience of burnout.

**Hypothesis** **5.** The relationship between resources and burnout is mediated by stress.

As for the relationship between health-promoting leadership and burnout, the mediating role of resources and stress was taken into account. Burnout is the result of an imbalance of stress and resources [23]; therefore, facilitating resources at work is crucial to cope with demands and lower stress. Health-promoting leadership aims to change working conditions in a way that employees experience more resources to cope with high demands. Experiencing more resources reduces stress and—in the long term—reduces burnout. The mediating roles of resources and stress are stated in following hypotheses:

**Hypothesis** **6.** The relationship between health-promoting leadership and stress is mediated by resources.

**Hypothesis** **7.** The relationship between health-promoting leadership and burnout is mediated by resources and stress.

**Figure 1:**
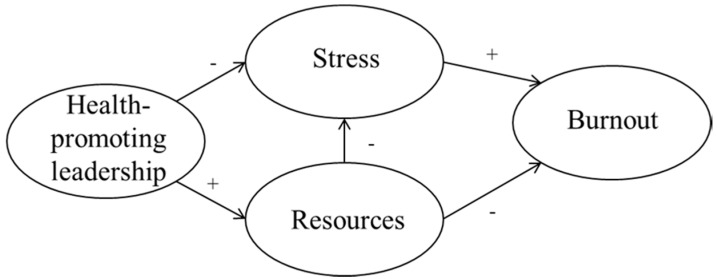
Hypothesized Model.

The hypothesized model is illustrated in Figure 1.

## 2. Materials and Methods 

### 2.1. Participants and Procedure

This study comprised two samples for two separate analysis steps. In the first sample, Austrian workers were invited to participate in an online study. The participants were invited in cooperation with a well-known German market research company by sending out e-mails. The participants had to fulfill the requirement of currently having a job; otherwise they were excluded at the beginning of the survey. The sample consisted of 228 participants who filled-in all questionnaires. In this sample, 47.8% were female and 52.2% were male. The participants were 30 years or younger (17.5%), 31 to 40 years old (28.5%), 41 to 50 years old (32%) and older than 50 years old (22%). Most of the participants worked full-time (40 h per week: 46.5%) or more than full-time (more than 40 h per week: 30.3%); the rest (23.2%) worked part-time. They worked in different business sectors, mostly in the business sectors commerce (18%), manufacturing (8.8%), public sector (8.8%), consulting (8.8%), and health care (8.3%). 

Similar to the first sample, the second sample consisted of participants working in Austria who were invited to participate in an online study. Invitations to the online study were spread on social platforms (e.g., Xing), and additionally distributed with the mailing list of a local human resource development network. Participants not fulfilling the requirement of currently having a job were excluded at the beginning of the study. All-in-all, 263 participants filled-in all questionnaires in this online study. Of these 263 participants, 68.6% were female and 31.4% were male. The participants were 30 years or younger (42.3%), 31 to 40 years old (27.1%), 41 to 50 years old (16.5%) and older than 50 years old (14.1%). About 40.4% of the participants worked full-time (40 h per week) and 27.1% worked more than 40 h per week. The rest (32.5%) worked part-time. The participants worked in various business sectors, mostly in health care (16.1%) education (15.3%), manufacturing (14.9%), and commerce (9.8%). 

### 2.2. Measures

#### 2.2.1. Health-Promoting Leadership Conditions (HPLC)

This questionnaire [27] measures the behavior of leaders in the last 4 weeks from the employees’ point of view. It includes seven dimensions of health-promoting leadership: health awareness, workload, control, reward, community, fairness and value-fit. Every dimension has three items which makes 21 items in total. One example item for the dimension health awareness is “In the last 4 weeks my leader took care that… the health of the employees is highly valued” and for the dimension reward “In the last 4 weeks my leader took care that… work is appreciated”. A seven point likert scale from zero (never) till six (always) is used. 

#### 2.2.2. Recovery-Stress-Questionnaire for Work (RESTQ-Work)

The Recovery-Stress-Questionnaire for Work (RESTQ-Work/55, [42]) has 55 items which address different aspects of stress and recovery/resources during the preceding seven days/nights. In the RESTQ-Work, recovery/resources are used interchangeably, so we use from now on the term resources. The items can be assigned to seven different sub-dimensions: social emotional stress, performance related stress, loss of meaning, overall recovery, leisure/breaks, psychosocial resources, and work related resources. These dimensions can be further classified into two scores: stress and resources. The items related to resources specifically measure resources at the workplace, which are related to recovery processes [31]. For the resources score one example item is ”In the past seven days/nights… I was able to relax during my breaks” or “In the past 7 days/nights… I had the chance to work on a variety of tasks”. One example item for a stress-related item is “In the past seven days/nights… I felt frustrated through my work”. The answer scale ranges from zero (never) to six (always). 

#### 2.2.3. Maslach Burnout Inventory—General Survey (MBI-GS-D)

The MBI-GS [43] measures burnout with three dimensions: emotional exhaustion, cynicism and personal accomplishment. In this study, only the core dimensions of burnout—emotional exhaustion and cynicism—were used. One example item for emotional exhaustion is “I feel emotionally drained by my job”. The participants got the German version of the MBI, the MBI-GS-D, by Büssing and Glaser [44] and were asked to answer the 10 items on a 6-point Likert scale ranging from one (never) to six (very often).

### 2.3. Analysis

For hypotheses testing, a confirmatory factor analysis (CFA) was calculated using the method of structural equation modelling (SEM) with maximum likelihood method of estimation. Health-promoting leadership, resources, stress and burnout were included as latent factors, operationalized by their assigned dimensions. In order to simplify the structure of the model, item parcels instead of items were entered as manifest variables in the model. Building item parcels can be a good approach to reduce unshared variance between the items that leads to correlated residuals [45]. The item parcels were created according to the underlying theoretical structure of the measurements. For example, health-promoting leadership was measured with the HPLC that builds upon seven dimension. Each dimension consists of three items which were aggregated into a parcel to represent the respective dimension. Thus, each parcel was built by calculating the means of the items representing one dimension.

SEM was used as a combination of confirmatory and exploratory testing by conducting the model assessment in the first sample and then verifying the findings in a second model assessment in the second sample. First, sample 1 was analyzed by testing the hypothesized structural model and modifying the model if needed. As post hoc modifications are prone to criticism (where one can argue that the model is just fitted to the existing data and not representing the reality), the model was cross validated with sample 2. 

As the concepts in the present study are multidimensional, the boundaries between these concepts might become vague. Therefore, we decided to remove overlapping constructs for the SEM. Removing the overlapping constructs was done on theoretical basis. The MBI-GS-D and the RESTQ-Work include components of exhaustion (operationalized in the RESTQ-Work with “loss of meaning”). As for the HPLC and the dimension resources of the RESTQ-Work, both questionnaires address social interactions (dimension community in the HPLC) as well as latitude (dimension control in the HPLC). These overlaps might lead to inflated correlations and thus the results could be difficult to interpret. Therefore, the overlapping constructs (loss of meaning in the RESTQ-Work, community, and control in the HPLC) were removed in the two samples.

Before conducting the SEM, measurement models were tested for both samples. The fit indices of the measurement models are in acceptable ranges. (AGFI: 0.875–0.930, GFI: 0.967–0.995, CFI: 0.965–0.999, RMSEA: 0.037–0.142). The RMSEA exceeded 0.10 in two measurement models (measurement model for resources in the first sample and measurement model for the HPLC in the second sample). The other fit indices of these models were in acceptable range. The descriptive statistic was analyzed with SPPS 21.0 (IBM, Armonk, NY, USA); the CFA was conducted using AMOS 21.0 (IBM, Armonk, NY, USA). 

## 3. Results

### 3.1. Descriptive Analysis

Descriptive statistics (means and standard deviations) of the study variables and their intercorrelations are shown in Table 1. The descriptive statistics were calculated for both samples separately. 

### 3.2. Structural Equation Modeling (SEM)

#### 3.2.1. Model Assessment in Sample 1 (N = 228)

**Figure 2:**
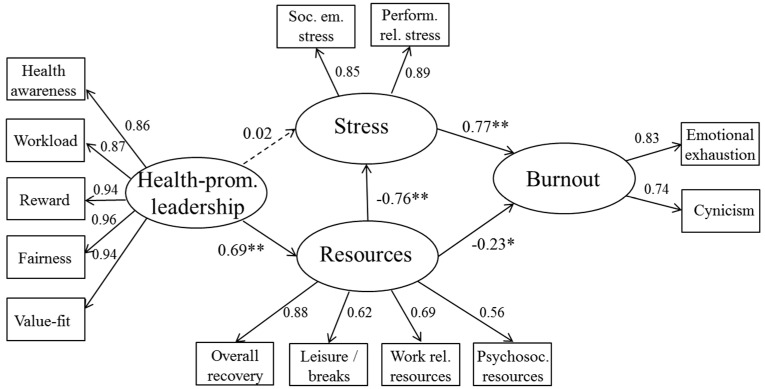
Structural Equation Modeling (SEM)—sample 1. ** paths are significant at *p* < 0.001; * paths are significant at *p* < 0.05.

The hypothesized model showed an acceptable fit (χ^2^ (56) = 162.960, *p* < 0.0001; AGFI = 0.846, GFI = 0.905, CFI = 0.958, RMSEA = 0.092). The model showed the predicted paths to be in the expected direction, with the exception of health-promoting leadership x stress (β = 0.02), which was not significant. As expected, health-promoting leadership shows a positive relationship with resources (β = 0.69) and resources is negatively related to stress (β = −0.76) and to burnout (β = −0.23). Stress shows a positive relationship with burnout (β = 0.77). The structural model with the regression paths is depicted in Figure 2. The direct, indirect and total effects as well as their confidence intervals are presented in Table 2, Table 3 and Table 4.

#### 3.2.2. Model Assessment in Sample 2 (N = 263)

**Figure 3:**
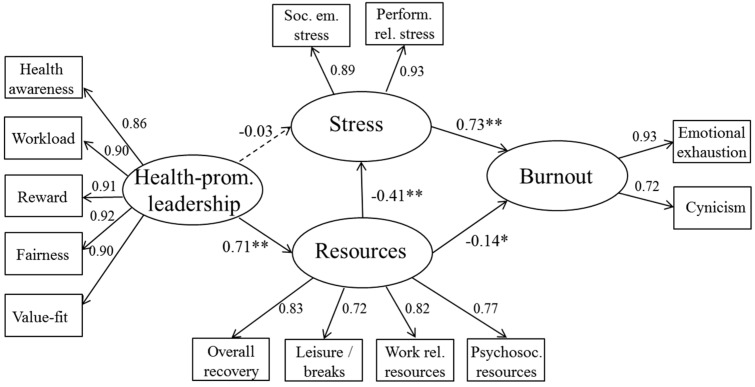
Structural Equation Modeling (SEM)—sample 2. ** paths are significant at *p* < 0.001; * paths are significant at *p* < 0.05.

In order to prove the plausibility of the model found in sample 1, the model has been cross validated with sample 2. The model (χ²(56) = 181.738, *p* < 0.0001) shows an acceptable fit (AGFI = 0.840, GFI = 0.902, CFI = 0.956, RMSEA = 0.093).The regression coefficients in this sample go in the same directions as in sample 1. Health-promoting leadership has a strong positive relationship with resources (β = 0.71), resources are negatively related to stress (β = −0.41) and burnout (β = −0.14). Stress shows a strong relationship with burnout (β = 0.73). The path between health-promoting leadership and stress (β = −0.03) was statistically not significant. The structural model of sample 2 is depicted in Figure 3. The direct, indirect and total effects as well as their confidence intervals are presented in Table 5, Table 6 and Table 7.

#### 3.2.3. Model Comparisons

Our hypothesized model assumed full mediation of resources and stress. More specifically, we assumed that the relationship between health-promoting leadership and burnout is completely explained by stress and resources. In order to verify that the full mediation model is the best model to fit the data, it has to be compared to an alternative, partial mediation model. In this partial mediation model, a direct path from health-promoting leadership to burnout was added. Although this model also provided a good fit to the data in sample 1 and sample 2, it did not provide a better fit compared to the full mediation model (Table 8). The direct paths from health-promoting leadership to burnout were not significant (Table 8).

## 4. Discussion

The present study aimed to investigate the paths between health-promoting leadership, resources, stress and burnout. The results support a model in which health-promoting leadership is related to the resources of employees. Resources in turn reduce stress and burnout. This model was cross-validated with a second sample and could be verified. The findings of this study contribute to the enrichment of the growing research on health-promoting leadership, and helps to clarify the contribution of resources and stress in the relationship between health-promoting leadership and the employees’ burnout.

We hypothesized that a high level of health-promoting leadership is positively related to resources and negatively related to stress. This hypothesis could only be partially verified. The findings support a model where health-promoting leadership has a strong positive connection to the employees’ resources, but the relationship between health-promoting leadership and employee stress is fully mediated by resources. Looking at the concept of health-promoting leadership, the essential part lies in influencing the conditions at the workplace by the leaders. Leaders enhance their employees’ resources by providing resource-oriented working conditions for their employees to support their health. More specifically, the leaders are enhancing the employees’ resources by taking responsibility for the employees’ health, raising the latitude, giving adequate reward, building a civil community, being fair as well as showing respect for the employees’ personal values. The strong relationship between health-promoting leadership and resources is also in line with previous works [14], where leaders impact their employees’ health by influencing job resources. 

The relationship between health-promoting leadership and stress was not significant in the model. The bivariate correlations are significant, though. This indicates that on a superficial level, health-promoting leadership behavior can reduce employee stress. Referring to models that take the balance between stress and resources into account [21,23], precursors and outcomes of stress should not be examined without considering resources. Our findings support the importance of including resources in research about leadership behavior, stress and burnout. Another explanation for not finding a direct relationship between health-promoting leadership and stress could lie in the operationalization of stress, which was measured as the subjective state of the person. Leaders engaging in health-promoting leadership aim at changing working conditions—which means changing the external working environment rather than changing the subjective reaction of their employees. 

However, one aspect of health-promoting leadership that specifically aims at reducing employee stress is the dimension of workload. Keeping workload at a tolerable level reduces the employees’ stress state and long-term health-related outcomes [46]. This can also be seen in the present study as low workload shows the highest negative correlations with the stress-related dimensions but also high positive correlations with the resources-related dimensions of the RESTQ-Work. 

We expected that the relationship between health-promoting leadership and burnout is fully mediated by resources and stress, which could be verified in the present findings. Comparing the full mediation model to a partial mediation model, we found that the partial mediation model did not significantly perform better than the full mediation model. This result suggests that the link between health-promoting leadership and burnout is completely explained by stress and resources. The absence of a direct link between health-promotion leadership and burnout, as well as between health-promotion leadership and stress could indicate that a stress- and burnout-reducing effect of health-promoting leadership is less likely unless the employees’ resources have been increased. 

Like in previous research [21,23], the results found in our model indicate that high resources are connected to lower stress. The direct path between resources and burnout seems to be low and even decreases further when health-promoting leadership is added as a direct predictor to burnout. The results indicate that stress is an important mediator in the relationship between resources and burnout. 

The path between stress and burnout is very high, which is in line with previous research [1,47]. However, both constructs can be clearly distinguished. Although exhaustion is an important aspect in current burnout models [43,47], it can be separated from stress, as stress can be seen as the precursor to emotional exhaustion [48]. 

According to our concept of health-promoting leadership, a leader that engages in health-promoting leadership supports employees by creating working conditions that lead to experiencing more resources, which in turn reduces stress and burnout. Our findings are consistent with the proposed concept: The models found in our study indicate that the relationship between health-promoting leadership and burnout is mediated by the employees’ resources and stress. The results of this study extend theory and previous research by showing that leaders engaging in health-promoting leadership do not synchronously lower stress and strengthen resources. Past research concluded that leadership behavior impacts resources more than it impacts stress [11]. Our findings indicate that a reduction of stress with leadership is still possible, although a stress-reducing effect might be more likely if leaders enhance resources, first. In line with our findings, we suggest focusing more strongly on the balance between the employees’ resources and stress as an important mediator in research about health-promoting leadership and health-related outcomes. 

At this point, it seems that health-promoting leadership can be a key element for the development of a healthy workplace by increasing the employees’ resources. This can be done either directly by changing working conditions in a way that employees see them as resources or by giving possibilities to recover and therefore restore lost resources. The approach to strengthen resources at the workplace to cope with job demands is also a more sustainable approach to change employee health in the current view of workplace health promotion [49].

As with any study, there are limitations to consider. In our tested model, we investigated the relationship between resources, stress, and burnout. We defined stress as the inner state of the person, which is caused as a reaction to external stressors [23]. To obtain a more complete model, stressors (i.e., the external influence aspects on the person) have to be included as well. Future studies should add stressors to the model to get more insight into the mechanisms between leadership and employee burnout. Cross-sectional online data was used to test the hypothesized relationships. Especially in case of mediation effects, causal interpretations must be done with caution. Online surveys have the advantage of reaching a large number of participants, which proved to be also a main advantage in the present study. A drawback of using online data is not having possibilities to check under which circumstances the questionnaires were answered. Furthermore, online surveys could raise the risk of getting a selected sample. We tried to avoid this problem by exploring two different samples—one sample from an online panel and the other from an open-access online survey. Having a look at the demographics of both samples, we see that both samples are well distributed. An additional limitation concerns common method bias. We applied some procedures suggested by Podsakoff et al. [50] to reduce common method bias: in the instruction, we highlighted the strict anonymity of the answers and asked the respondents to answer the questions as honestly as possible. Separating measurement of predictor and criterion variable was not possible, as the online survey had to be filled-in without larger breaks to prevent potential intervening factors between measuring predictor and criterion variable. In the survey, the answer times were logged and analyzed. Data with suspicious answer times were removed prior to the analyses. 

## 5. Conclusions

The indirect effect of health-promoting leadership on employee health by changing working conditions shows to be a good approach for leaders to support their employees’ health at the workplace. Leaders engaging in health-promoting leadership support their employees’ health by creating health-supportive working conditions that enhances resources at the workplace, which in turn reduces stress and burnout.

Another important result of our study is the direct path of health-promoting leadership on resources and the indirect effect on stress. In sample 2, we can confirm this result. Enhancing resources leads to reducing stress and therefore reducing burnout. Furthermore, the impairing effects of stress (e.g., burnout) can be reduced by raising resources. In today’s working environment, reducing stress by keeping workload at a moderate level is not always possible and might even be unfitting for certain tasks or jobs (e.g., very demanding jobs such as hospital work). Building up resources might be a more suitable approach in the practical field for leaders to support their employees dealing with high job demands. 

Leaders can be a success factor for a better working environment. However, that does not mean to focus (only) on changing the behavior of leaders via leadership trainings, but more to look at the working conditions. Trainings should support leaders in improving working conditions to support a better workplace. The boundaries of these health-promoting behaviors of leaders lie in the options the leaders have in their work environment, which is also underlined by Rigotti et al. [11]. This again strengthens the point that leaders have to be supported to be supportive. Therefore, even more importantly, the organizational culture should give the leaders a wide range of possibilities to influence the working environment. 

Future research should focus on identifying the causal relationship between health-promoting leadership, stress, resources and burnout. Longitudinal studies over longer time periods are needed to test causal effects. Furthermore, the model should be verified in big samples to test the stability of the model. In addition, future studies should investigate barriers and facilitators that can hinder or support leaders in engaging in health-promoting leadership behavior. The organization has to create an appropriate framework for their leaders to be able to change the working conditions with their leadership behavior.

## Figures and Tables

**Table 1:** Means, standard deviation, Cronbach’s Alpha (α) and correlations between all study variables (sample 1 and sample 2).

No.	Dimension	M	SD	α	M	SD	α	1	2	3	4	5	6	7	8	9	10	11	12	13	14	15	16
1	Health awareness *(HPLC)*	2.32	1.87	*0.95*	*2.72*	*1.85*	0.94	-	0.82 **	0.69 **	0.77 **	0.72 **	0.77 **	0.75 **	0.43 **	0.48 * *	0.54 **	0.47 **	−0.26 **	−0.26 **	−0.38 **	−0.28 **	−0.37 **
2	Workload *(HPLC)*	2.59	1.68	*0.86*	*2.66*	*1.70*	0.88	0.79 **	-	0.78 **	0.81 **	0.74 **	0.81 **	0.80 **	0.47 **	0.56 **	0.58 **	0.45 **	−0.31 **	−0.34 **	−0.44 **	−0.33 **	−0.37 **
3	Control *(HPLC)*	2.95	1.75	*0.91*	*3.47*	*1.67*	0.88	0.74 **	0.81 **	-	0.81 **	0.71 **	0.81 **	0.81 **	0.52 **	0.49 **	0.72 **	0.56 **	−0.30 **	−0.24 **	−0.37 **	−0.26 **	−0.40 **
4	Reward *(HPLC)*	2.61	1.82	*0.94*	*3.06*	*1.75*	0.91	0.80 **	0.81 **	0.86 **	-	0.78 **	0.85 **	0.82 **	0.48 **	0.44 **	0.64 **	0.49 **	−0.26 **	−0.26 **	−0.41 **	−0.29 **	−0.43 **
5	Community *(HPLC)*	3.06	1.93	*0.95*	*3.47*	*1.87*	0.93	0.75 **	0.75 **	0.85 **	0.84 **	-	0.84 **	0.77 **	0.45 **	0.41 **	0.58 **	0.57 **	−0.24 **	−0.24 **	−0.40 **	−0.25 **	−0.38 **
6	Fairness *(HPLC)*	2.75	1.75	*0.87*	*3.32*	*1.80*	0.88	0.80 **	0.83 **	0.89 **	0.91 **	0.87 **	-	0.83 **	0.46 **	0.44 **	0.64 **	0.52 **	−0.27 **	−0.24 **	−0.38 **	−0.27 **	−0.41 **
7	Value-fit *(HPLC)*	2.55	1.78	*0.91*	*3.12*	*1.70*	0.87	0.82 **	0.80 **	0.85 **	0.88 **	0.79 **	0.89 **	-	0.53 **	0.46 **	0.67 **	0.54 **	−0.26 **	−0.23 **	−0.37 **	−0.27 **	−0.40 **
8	Overall recovery *(RESTQ-W)*	3.44	1.01	*0.86*	*3.33*	*1.13*	0.90	0.43 **	0.45 **	0.49 **	0.55 **	0.50 **	0.52 **	0.50 **	-	0.60 **	0.69 **	0.67 **	−0.38 **	−0.38 **	−0.41 **	−0.37 **	−0.34 **
9	Leisure/breaks *(RESTQ-W*)	3.22	1.19	*0.87*	*3.26*	*1.24*	0.85	0.39 **	0.45* *	0.37 **	0.38 **	0.37 **	0.40 **	0.36 **	0.55 **	-	0.52 **	0.55 **	−0.45 **	−0.52 **	−0.56 **	−0.45 **	−0.33 **
10	Work related resources *(RESTQ-W)*	3.29	1.36	*0.89*	*3.39*	*1.46*	0.93	0.52 **	0.52 **	0.66 **	0.63 **	0.56 **	0.62 **	0.63 **	0.62 **	0.30 **	-	0.65 **	−0.25 **	−0.24 **	−0.34 **	−0.28 **	−0.46 **
11	Psychosocial resources *(RESTQ-W)*	3.60	1.45	*0.85*	*3.87*	*1.44*	0.85	0.38 **	0.41**	0.42 **	0.42 **	0.52 **	0.42 **	0.36 **	0.50 **	0.39 **	0.43 **	-	−0.20 **	−0.18 **	−0.28 **	−0.18 **	−0.28 **
12	Social emotional stress *(RESTQ-W)*	2.20	1.18	*0.90*	*2.03*	*1.21*	0.92	−0.37 **	−0.39 **	−0.42 **	−0.43 **	−0.38 **	−0.45 **	−0.42 **	−0.57 **	−0.46 **	−0.34 **	−0.24 **	-	0.83 **	0.80 **	0.64 **	0.50 **
13	Performance related stress *(RESTQ-W)*	2.12	1.11	*0.86*	*2.02*	*1.13*	0.88	−0.36 **	−0.38 **	−0.40 **	−0.36 **	−0.34 **	−0.42 **	−0.39 **	−0.63 **	−0.58 **	−0.36 **	−0.26 **	0.77 **	–	0.83 **	0.70 **	0.47 **
14	Loss of meaning *(RESTQ-W)*	1.83	1.13	*0.91*	*1.72*	*1.13*	0.92	−0.42 **	−0.44 **	−0.47 **	−0.47 **	−0.46 **	−0.51 **	−0.45 **	−0.61 **	−0.47 **	−0.41 **	−0.29 **	0.78 **	0.79 **	–	0.73 **	0.61 **
15	Emotional Exhaustion *(MBI-GS-D)*	3.46	1.18	*0.90*	*3.07*	*1.20*	0.90	−0.39 **	−0.48 **	−0.38 **	−0.41 **	−0.35 **	−0.40 **	−0.38 **	−0.59 **	−0.51 **	−0.37 **	−0.24 **	0.65 **	0.72 **	0.72 **	-	0.66 **
16	Cynicism *(MBI-GS-D)*	2.98	1.26	*0.87*	*2.49*	*1.13*	0.87	−0.46 **	−0.45 **	−0.54 **	−0.54 **	−0.51 **	−0.56 **	−0.55 **	−0.57 **	−0.30 **	−0.59 **	−0.28 **	0.58 **	0.56 **	0.68 **	0.59 **	-

Note: Correlations are significant at ** *p* < 0.01 (two-tailed); normal type, lower left part: sample 1 (N = 228), italic type, upper right part: sample 2 (N = 263); answer scales: HPLC 0 (never) to 6 (always), RESTQ-W 0 (never) to 6 (always), MBI-GS-D 1 (never) to 6 (very often).

**Table 2:** Direct impacts in the structural model—sample 1.

Paths	Direct Impact	Sig.	95% CI
Health-prom. leadership → Stress	0.021	ns.	[−0.165; 0.214]
Health-prom. leadership → Resources	0.688	*p* < 0.001	[−0.585; 0.778]
Health-prom. leadership → Burnout	0.000	ns.	[0.000; 0.000]
Resources → Stress	−0.762	*p* < 0.001	[−0.934; −0.597]
Resources → Burnout	−0.225	0.009	[−0.413; −0.061]
Stress → Burnout	0.766	*p* < 0.001	[0.602; 0.926]

Note: ns.: not significant.

**Table 3:** Indirect impacts in the structural model—sample 1.

Paths	Indirect Impact	Sig.	95% CI
Health-prom. leadership → Stress	−0.524	*p* < 0.001	[−0.687; −0.393]
Health-prom. leadership → Resources	n.a	n.a	n.a
Health-prom. leadership → Burnout	−0.540	*p* < 0.001	[−0.657; −0.434]
Resources → Stress	n.a	n.a	n.a
Resources → Burnout	−0.583	*p* < 0.001	[−0.766; −0.428]
Stress → Burnout	n.a	n.a	n.a

Note: n.a: not applicable; ns.: not significant.

**Table 4:** Total impacts in the structural model—sample 1.

Paths	Total impact	Sig.	95% CI
Health-prom. leadership → Stress	−0.503	*p* < 0.001	[−0.606; −0.397]
Health-prom. leadership → Resources	0.688	*p* < 0.001	[0.585; 0.778]
Health-prom. leadership → Burnout	−0.540	*p* < 0.001	[−0.657; −0.434]
Resources → Stress	−0.762	*p* < 0.001	[−0.934; −0.597]
Resources → Burnout	−0.808	*p* < 0.001	[−0.959; −0.638]
Stress → Burnout	0.766	*p* < 0.001	[0.602; 0.926]

Note: ns.: not significant.

**Table 5:** Direct impacts in the structural model—sample 2.

Paths	Direct Impact	Sig.	95% CI
Health-prom. leadership → Stress	−0.034	ns.	[−0.236; 0.110]
Health-prom. leadership → Resources	0.713	*p* < 0.001	[0.582; 0.794]
Health-prom. leadership → Burnout	0.000	ns.	[0.000; 0.000]
Resources → Stress	−0.410	*p* < 0.001	[−0.582; −0.244]
Resources → Burnout	−0.142	0.009	[−0.287; −0.029]
Stress → Burnout	0.725	*p* < 0.001	[0.625; 0.817]

Note: ns.: not significant.

**Table 6:** Indirect impacts in the structural model—sample 2.

Paths	Direct Impact	Sig.	95% CI
Health-prom. leadership → Stress	−0.292	*p* < 0.001	[−0.432; −0.173]
Health-prom. leadership → Resources	n.a	n.a	n.a
Health-prom. leadership → Burnout	−0.338	*p* < 0.001	[−0.489; −0.209]
Resources → Stress	n.a	n.a	n.a
Resources → Burnout	−0.297	*p* < 0.001	[−0.419; −0.174]
Stress → Burnout	n.a	n.a	n.a

Note: n.a: not applicable; ns.: not significant.

**Table 7:** Total impacts in the structural model—sample 2.

Paths	Direct Impact	Sig.	95% CI
Health-prom. leadership → Stress	−0.326	*p* < 0.001	[−0.476; −0.168]
Health-prom. leadership → Resources	0.713	*p* < 0.001	[0.582; 0.794]
Health-prom. Leadership → Burnout	−0.338	*p* < 0.001	[−0.489; −0.209]
Resources → Stress	−0.410	*p* < 0.001	[−0.582; −0.244]
Resources → Burnout	−0.439	*p* < 0.001	[−0.630; −0.277]
Stress → Burnout	0.725	*p* < 0.001	[0.625; 0.817]

Note: ns.: not significant.

**Table 8:** Fit indices and path coefficients of the full mediation model and partial mediation model.

Fit indices and Regression Paths	Full Mediation Model—Sample 1	Full Mediation Model—Sample 2	Partial Mediation Model—Sample 1	Partial Mediation Model—Sample 2
Chi2	162.960	181.738	161.903	180.973
df	56	56	55	55
p	<0.001	<0.001	<0.001	<0.001
AGFI	0.846	0.840	0.844	0.838
GFI	0.905	0.902	0.905	0.902
CFI	0.958	0.956	0.958	0.956
RMSEA	0.092	0.093	0.093	0.093
Health-prom. leadership → Stress	0.021	−0.034	0.033	−0.026
Health-prom. leadership → Resources	0.688 **	0.713 **	0.684 **	0.711 **
Health-prom. leadership → Burnout	-	-	−0.073	−0.066
Resources → Stress	−0.762 **	−0.410 **	−0.771 **	−0.417 **
Resources -> Burnout	−0.225 *	−0.142 *	−0.162	−0.091
Stress -> Burnout	0.766 **	0.725 **	0.777 **	0.729 **

Note: ** paths are significant at *p* < 0.001; * paths are significant at *p* < 0.05.
